# Locus Coeruleus Optogenetic Light Activation Induces Long-Term Potentiation of Perforant Path Population Spike Amplitude in Rat Dentate Gyrus

**DOI:** 10.3389/fnsys.2018.00067

**Published:** 2019-01-09

**Authors:** Meghan A. L. Quinlan, Vanessa M. Strong, Darlene M. Skinner, Gerard M. Martin, Carolyn W. Harley, Susan G. Walling

**Affiliations:** Behavioural Neuroscience Group, Department of Psychology, Memorial University of Newfoundland, St. John’s, NL, Canada

**Keywords:** locus coeruleus, dentate gyrus, long-term potentiation, short-term potentiation, norepinephrine, perforant path, hippocampus, optogenetic

## Abstract

Norepinephrine (NE) in dentate gyrus (DG) produces NE-dependent long-term potentiation (NE-LTP) of the perforant path-evoked potential population spike both *in vitro* and *in vivo*. Chemical activators infused near locus coeruleus (LC), the source of DG NE, produce a NE-LTP that is associative, i.e., requires concurrent pairing with perforant path (PP) input. Here, we ask if LC optogenetic stimulation that allows us to activate only LC neurons can induce NE-LTP in DG. We use an adeno-associated viral vector containing a depolarizing channel (AAV8-Ef1a-DIO-eChR2(h134r)-EYFP-WPRE) infused stereotaxically into the LC of TH:Cre rats to produce light-sensitive LC neurons. A co-localization of ~62% in LC neurons was observed for these channels. Under urethane anesthesia, we demonstrated that 5–10 s 10 Hz trains of 30 ms light pulses in LC reliably activated neurons near an LC optoprobe. Ten minutes of the same train paired with 0.1 Hz PP electrical stimulation produced a delayed NE-LTP of population spike amplitude, but not EPSP slope. A leftward shift in the population spike input/output curve at the end of the experiment was also consistent with long-term population spike potentiation. LC neuron activity during the 10 min light train was unexpectedly transient. Increased LC neuronal firing was seen only for the first 2 min of the light train. NE-LTP was more delayed and less robust than reported with LC chemo-activation. Previous estimates of LC axonal conduction times suggest acute release of NE occurs 40–70 ms after an LC neuron action potential. We used single LC light pulses to examine acute effects of NE release and found potentiated population spike amplitude when a light pulse in LC occurred 40–50 ms, but not 20–30 ms, prior to a PP pulse, consistent with conduction estimates. These effects of LC optogenetic activation reinforce evidence for a continuum of NE potentiation effects in DG. The single pulse effects mirror an earlier report using LC electrical stimulation. These acute effects support an attentional role of LC activation. The LTP of PP responses induced by optogenetic LC activation is consistent with the role of LC in long-term learning and memory.

## Introduction

The locus coeruleus (LC) has long been implicated in learning and memory and has recently been highlighted as a possible ground zero for Alzheimer’s Disease, a memory pathway selective disease (Braak et al., 2011; Braak and Del Tredici, 2015). One of the changes in learning that is strongly associated with aging (Dillon et al., 2017; Leal and Yassa, 2018) and with LC function (Segal et al., 2012; Bekinschtein et al., 2013) is pattern separation dependent on the dentate gyrus (DG). There is evidence for a special role of norepinephrine (NE), the neurotransmitter released from LC neurons, in network circuit changes in DG. In particular, NE-long term potentiation (NE-LTP) of perforant path (PP) input has been repeatedly demonstrated *in vitro* (Lacaille and Harley, 1985; Stanton and Sarvey, 1985, 1987) and *in vivo* (Neuman and Harley, 1983; Harley and Milway, 1986; Harley et al., 1989; Walling and Harley, 2004) in this structure and, more recently, NE-long term depression (NE-LTD; Hansen and Manahan-Vaughan, 2015b) has also been reported. Here, we use optogenetic methods to examine the influence of LC activation on plasticity of PP input to the DG.

The first examination of NE effects on PP-evoked potential in rat DG used iontophoretic NE (Neuman and Harley, 1983) and found both transient, and enduring, population spike potentiation, while the population EPSP slope was rarely increased. NE-population spike potentiation was reproduced *in vitro* and was β-adrenergic receptor dependent (Lacaille and Harley, 1985; Stanton and Sarvey, 1985). Later experiments demonstrated that glutamatergic (Walling and Harley, 2004) and orexinergic activation of the LC (Walling et al., 2004), evoking natural release of NE in DG, produces both transient and long-lasting PP-spike potentiation that is β-adrenergic-dependent. While most *in vivo* experiments were conducted under urethane-anesthesia, the same pattern of results is seen with glutamatergic LC activation in awake rats and NE-spike potentiation can last up to 24 h (Walling et al., 2004).

More recently, Hansen and Manahan-Vaughan (2015b) have reported 1 s of 100 Hz LC-electrical stimulation induces, not long-term NE-LTP, but long-term NE-LTD of the PP-evoked potential in DG. Both population EPSP slope and spike were depressed and these changes lasted up to 24 h in their awake rats. The authors note, however, that an earlier study using brief 50 Hz LC electrical stimulation trains in anesthetized rats (Dahl and Winson, 1985) produced, not depression, but potentiation of the PP-population spike without changes in the cell body EPSP slope as reported above for LC chemoactivation. They suggest the differences in outcome with LC activation are likely related to LC electrical stimulation frequencies.

The chemoactivation of LC that appears reliably linked to population spike potentiation provides some degree of spatial selectivity for LC. It does not activate axons of passage, but provides no firm control of firing frequencies and depending on the spread of infusions may also not be spatially selective. Electrical stimulation, in contrast, offers the ability to alter both the strength and frequencies of stimulation, but neither is easily related to LC firing patterns and is always likely to recruit axonal fibers of passage before LC cell bodies.

Here, we use optogenetic methods to selectively activate only LC neurons. We employ temporal parameters that we can demonstrate drive LC firing. We ask whether such activation produces PP-population spike potentiation or depression.

## Materials and Methods

### Animals

Four male and 17 female TH:Cre Sprague-Dawley rats bred from homozygous male TH:Cre rats (Sage Laboratories, MA, USA) and wild-type Sprague-Dawley females (Charles River) were used. The rats were housed in individual cages on a reverse light-dark cycle, and received food and water *ad libitum*. This study was carried out in accordance with the recommendations of the Canadian Council on Animal Care. The protocol was approved by the Institutional Animal Care Committee.

### Surgery and Optogenetic Transduction

Rats were infused between 4 and 6 months of age with a viral vector and photoactivatable channel gene under isofluorane anesthesia. In each hemisphere two 0.5 μL infusions of an AAV8-Ef1a-DIO-eChR2(h134r)-EYFP-WPRE solution of ~5^12^ vg/ml were directed adjacent to the LC using a 33 ga cannula (Plastic Products). The cannula was lowered at an angle of 20° posterior to vertical (to avoid the transverse sinus) at 12.0 mm posterior to, and at both 1.2 mm and 1.3 mm lateral to, bregma to a depth of ~5.5 mm below brain surface. Infusions were made at 0.1 μL/min. The cannula was left in place for 5 min following each infusion. Thus a total of 1.0 μL per hemisphere was infused and both hemispheres received infusions. For 11 rats, in addition to the virus, 0.07 μL of fluorescent beads (1–5 μm; 407–445 nm excitation-emission spectrum; Cospheric, Santa Barbara, CA, USA) were infused at the same coordinates to mark the AAV infusion location. AAV infusion material and associated genes were generously provided by Dr. Karl Deisseroth (Stanford University, CA, USA).

### Locus Coeruleus Electrophysiology

Recordings were carried out 1.5 months to 8 months post-viral infusion. Rats were anesthetized with 15% urethane (1 ml/100 g), placed in a stereotaxic apparatus with skull flat and body temperature was maintained at 37°C. A skull screw placed in the contralateral hemisphere anterior to bregma served as ground and the stereotaxic apparatus was also grounded.

The prior LC opening was cleared for placement of an optrode assembly. Optrode assemblies were hand constructed prior to use using a 400 μm glass optic fiber (Thorlabs Inc, Newton, NJ, USA) aligned contiguously and posterior to a tungsten electrode (2–3 μm, 4 MΩ; FHC) with the electrode tip protruding ~50–100 μm ventral to the optic fiber. The optic fiber was connected to a 450 nm laser diode fiber light source (Doric Lenses) *via* a patch cord, which in turn was connected to a computer running Doric Lenses software to control light parameters. The optrode assembly was slowly lowered toward the LC (12.0–12.8 mm posterior depending on rat size and 1.3 mm lateral to bregma at a 20° angle) to 6.1–6.9 mm below brain surface until putative LC cells could be located. Putative LC neurons were identified by audio-monitor and oscilloscope using several criteria: slow spontaneous firing frequency, broad action potentials and a response to tail or toe pinch. Cells were recorded digitally using SciWorks (DataWave) with input digitized at 20 kHz and band pass filtered from 600 Hz-3 kHz. Data was collected in 10 s sweeps, with an average program processing time of 26 ms between sweeps.

To test light activation of LC neurons, light sequences of 10 Hz, 30 ms pulses of 150 mA light (60–70 mW of light as assessed in optrode tests) were applied for either 5 s or 10 s. Each light test was separated by at least 2 min. Ten Hz had previously been shown to be in an effective range for LC optogenetic activation with a similar channel (Carter et al., 2010) and corresponded to a firing frequency which LC neurons generate under naturalistic conditions linked to a high level of interest for an environmental stimulus (Foote et al., 1980).

### Perforant Path (PP)-DG Electrophysiology

Following confirmation of successful viral expression and light activation (see “Results” section), a subset of eight female rats were prepared to collect PP evoked potentials from the DG. For PP stimulation a hole was drilled 7.2 mm posterior to, and 4.1 mm lateral to, bregma and an NE-100 concentric bipolar electrode (Kopf) was directed at the PP ~3 mm below brain surface in the left hemisphere (in these rats putative LC neurons were located in the left hemisphere). For recording, a hole was drilled 3.5 mm posterior to, and 2 mm lateral from, bregma and a tungsten recording electrode (FHC) like that used for LC recording was lowered ~3–4 mm below brain surface.

The depth of the stimulating and recording electrodes were adjusted to maximize the PP-evoked potential population spike amplitude. The PP was stimulated with a 0.2 ms pulse every 10 s for the duration of the experiment. The responses from the recording electrode in DG were collected using SciWorks and the evoked potential was digitized at 10 kHz and band pass filtered from 1 Hz to 3 kHz.

The evoked potential protocol began with input/output stimulation. The PP was stimulated (0.2 ms pulse) over the range of 100–1,000 μA beginning with 100 and continuing in 100 μA steps. At each current level, two pulses were given at three different interstimulus interval (ISI), 30 ms, 70 ms and 200 ms. This permitted determination of an input/output curve for the first pulse while the 2nd pulse probed feedback network effects. A single pulse level was then chosen for the remainder of the experiment, either the current required for a 50% maximal population spike (four animals) or at the first current to give a maximal population spike (four animals). These two stimulation conditions were chosen because tetanic LTP studies normally use a 50% current, while previous NE-LTP studies have used a maximal current and we were interested in whether that choice would affect the percentage potentiation measured. Baseline PP stimulation continued until a stable 30 min had been collected. Then, LC light activation was given to verify an optical LC response, three 10 s trains (see “Locus Coeruleus Electrophysiology” section) separated by at least 2 min each were administered to verify effective activation. Following an additional 2 min without optical activation, a 10 min optical LC train was given. The 0.1 Hz PP stimulation continued throughout these manipulations. PP-evoked potentials were followed for 3 h after the putative 10 min of LC light activation. The 10 min activation protocol was selected based on our previous experience with 10 min of LC electrical stimulation given in bursts (Harley et al., 1989). In the 1989 study, we found the 10 min protocol produced a delayed long-lasting potentiation of PP-population spike amplitude similar in magnitude to that seen with glutamate activation of LC. We had also had extensive experience using a 10 min pairing of olfactory input and activation of LC-NE input to generate odor preference learning in rat pups (Yuan et al., 2014) suggesting that a 10 min period of LC activation might occur in effective naturalistic associative learning settings. In pilot studies for the present experiments, we had found that a 0.5 s train of LC light activation, unlike the glutamate activation protocol we use normally (Harley and Sara, 1992), was unable to initiate LTP effects, possibly due to more limited recruitment of LC neurons and/or to more modest depolarization effects.

At the end of the final 3 h of recording, another LC light subroutine was initiated, in which single LC pulses of either 30 ms or 50 ms duration were given followed 24 ms (the minimum permitted by DataWave programming tools), 30 ms, 40 ms or 50 ms later by a single PP stimulation. Each interval was sampled 10 times in a block. A pseudorandom order was chosen for these probe blocks (pulse width-ISI in ms: 50–50, 30–30, 50–24, 50–30, 30–24, 30–50, 30–40, 50–40.

These probes ascertained whether single LC optical pulses could modulate the DG evoked potential and, if so, was there an optimal latency. Finally, a second input output curve at the 10 current levels used initially with paired pulses at the same three ISI was administered.

### Perfusion and Immunohistochemistry

Rats were transcardially perfused with 0.9% saline and 4% paraformaldehyde. Brains were stored in refrigerated 4% paraformaldehyde for at least 24 h, and then refrigerated in 20% sucrose (made in 0.1 M phosphate buffer) for at least three days. Finally, brains were flash frozen in methyl butane, and stored in a −80°C freezer until processed. Brains were sectioned coronally at 30 μm on a Leica CM3050S cryostat and sections were stored in polyvinylpyrrolidone storage solution. Alternate sections were stained using a Nissl procedure for perfused tissue to verify placement of optrodes and electrodes. This procedure entailed dehydrating and rehydrating the tissue with ethanol (100%, 95% and 70%) and xylene, followed by a quick wash in distilled water and then 5 min in 1% cresyl violet. This was subsequently followed by washes in distilled water, 1% acetic acid and distilled water again, before a final series of dehydration steps and cover-slipping with Permount (FisherChemicals).

Using Nissl sections for localization, six LC, as well as two sections with the deepest probe placement, were selected for immunohistochemistry. Cells of the LC were visualized using a monoclonal antibody raised in mouse against dopamine- β-hydroxylase (DBH; Millipore, Burlington, MA, USA). Free-floating sections were washed with Tris buffer (0.1 M, pH 7.6) three times, at 5 min intervals to remove remaining fixative. They were transferred to new wells and washed with 1% hydrogen peroxide in Tris buffer for 30 min. Several washes followed, beginning with Tris for 5 min, Tris A (0.1% Triton X in Tris buffer) for 10 min and Tris B (0.1% Triton X + 0.005% BSA in Tris) for 10 min. The sections were next incubated for 1 h in 10% normal donkey serum (Jackson ImmunoResearch, PA, USA) diluted in Tris B, to bind non-specific antigens. Following the incubation period, sections were again washed in Tris A for 10 min and Tris B for 10 min, and were then incubated in primary antibody at a 1:10,000 dilution in Tris B (2–7 days, ~4°C). Next, sections were again washed in Tris A for 10 min and Tris B for 10 min. Sections were incubated in a 1:400 dilution of Alexa-555 donkey α-mouse (Invitrogen, Carlsbad, CA, USA) in Tris B for 1 h; and finally, washed twice in Tris buffer for 10 min consecutively. Sections were wet mounted onto dipped slides with Tris buffer and cover slipped using Vetashield antifading mounting medium with DAPI (Vector Labs). Localization of beads and optrodes was made on coronal sections and reconstructed using schematics adapted from coronal plates in a Paxinos and Watson atlas (Paxinos and Watson, 1998).

### Data analysis for Electrophysiology

#### LC Cell Responses

Analysis thresholds were set to analyze cells 1.5 × larger in amplitude than background. The relevant features of cell waveforms were extracted by DataWave autosort protocol based on six dimensions: peak time, peak amplitude, valley one amplitude, valley 2 (Brown et al., 2005) amplitude and two principal components. Unique cellular waveforms were then separated and assigned to their own cluster. Frequency histograms were completed within DataWave for each light trial and each unique waveform respectively. These histograms provided a count of cell firing per second with 1-min baselines collected both before and after the light protocol. Frequency histograms were generated to compare the firing rates of LC cells before, during and after each light protocol. A repeated measures analysis of variance (ANOVA) was performed to analyze differences in neuronal firing patterns before, during and after the light protocols. Paired samples *t*-tests were used to examine specific differences between the neuronal firing patterns just before and during the light protocols as well as in the time just following the light. Criterion was set to *p* < 0.05 for significance. While control experiments were not carried out here, we have previously published three articles with similar protocols and shown highly stable PP-DG evoked potential responses over 3 h post baseline monitoring for control groups (*n* = 7–10/rats/group; Walling and Harley, 2004; Walling et al., 2004; Lethbridge et al., 2014).

Putative LC cellular waveforms were analyzed for three parameters: amplitude (μV) defined as the voltage difference between the waveform’s valley (minimum point) and the second maximum; peak latency (μs) defined as the time between those two points; half width (μs) defined by the width of the spike half way between the maximum and minimum points; and asymmetry calculated as the ratio of amplitude of the second maximum point to the amplitude of the first maximum (Weir et al., 2014). These values were then averaged. The specific waveforms selected for inclusion in our analysis reflected at least two characteristics of LC cells: broad action potentials and slow firing rates of <6 Hz.

#### Evoked Potential Analysis

The PP-evoked potentials recorded in the DG that had been digitally stored were processed by DataWave analysis programs to calculate the population spike latency (ms), EPSP slope (mV/ms) and population spike amplitude (mV) of the PP-evoked potential for each potential. Five-minute averages were computed and graphed for each subject. Absolute values were then normalized as a percentage of the 30-min pre-light baseline. The normalized values were compared over time in repeated measure ANOVAs to determine if there was a change in evoked potential parameters in relation to the light protocol and whether any change later returned to the original baseline values. ANOVAs were also carried out on the input/output curves. The three sets of pre and post PP ISI data were analyzed with separate *t*-tests while the LC-PP ISI data were analyzed with ANOVAs. Results throughout are reported as means +/− standard error of the mean (SEM).

## Results

### Co-localization

**Figure 1:**
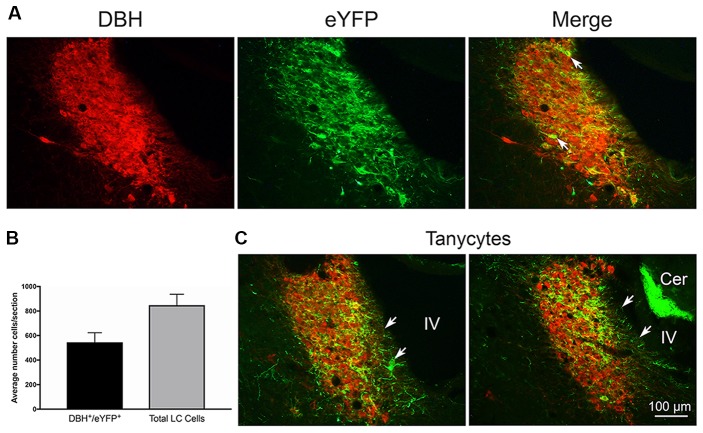
**(A)** Left: dopamine-β-hydroxylase (DBH) staining of locus coeruleus (LC) neurons. Middle: enhanced yellow fluorescent protein (eYFP) expression in TH:Cre LC neurons. Right: merge image of both markers. Arrow indicates an example of occlusion of DBH staining by strong eYFP expression in a TH:Cre neuron. **(B)** Graph of average co-localization of the channel expression marker eYFP^+^ in LC neurons sampled in three LC sections from 19 rats. **(C)** Coronal LC sections from two rats illustrating eYFP^+^ expression in putative tanycyte cells and processes at the medial ventricular non-neuronal LC margin. White arrows indicate tanycyte exemplars. Note another example of medial eYFP^+^ cells and processes in the merge panel in **(A)** under the white arrow stem. Cer = cerebellum with autofluorescing granule cells. IV = adjacent 4th ventricle.

Twenty-one rats were infused with the virus-channel combination. Eleven rats of those rats were also infused with beads, one with beads directly in the LC was discarded due to damage, and one did not have good channel expression. The left hemisphere LC was counted in three central sections to estimate co-localization. The total number of LC cells counted/section/rat on average was 848 ± 93 (SEM) and did not differ between bead and no-bead groups. Overall co-localization as measured by the proportion of eYFP expressing neurons within the LC borders, as defined by overall DBH reactivity, was 62.4%, see Figure 1 for examples of co-localization. We found eYFP expression could occlude DBH reactivity within the nucleus (white arrows in Figure 1A). Such eYFP occlusion has been observed in other laboratories (Hickey et al., 2014; Soya et al., 2017). There were no differences in channel expression between bead-infused and non bead-infused rats. Histology in bead-infused rats (*n* = 9) revealed beads located near the anterior border of LC. In depth, beads were typically ventral to LC (*n* = 5), but were also seen at the level of LC (*n* = 3) and dorsal to it (*n* = 1).

In a majority of rats, we also observed eYFP in processes that extended from the medial LC border to the wall of the IVth ventricle. There are no neuronal processes in this zone, thus the eYFP in these processes likely reflects transfer to, or expression of, channels in glia, probably tanycytes (Burnett and Felten, 1981; Felten et al., 1981; Cabral et al., 2013). We illustrate these observations in Figures 1A,C.

### Locus Coeruleus Optical Stimulation With 5 and 10 s light Trains

**Figure 2:**
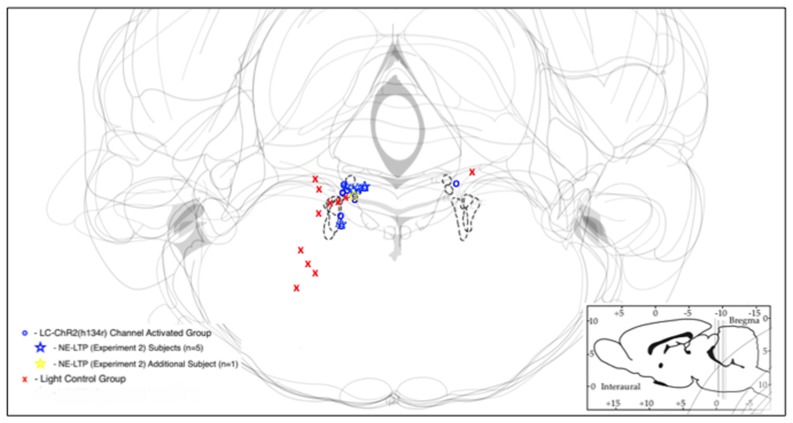
Schematic representations of LC with optrode placements designated by symbols. Blue/yellow symbols represent optrode placements that successfully produced light activation of the recorded cells. Red symbols indicate optrodes that did not produce light activation of recorded cells. Sites at which long-term potentiation (NE-LTP) experiments were conducted are indicated by stars. The dashed lines indicate the outlines of LC in each respective section.

**Figure 3:**
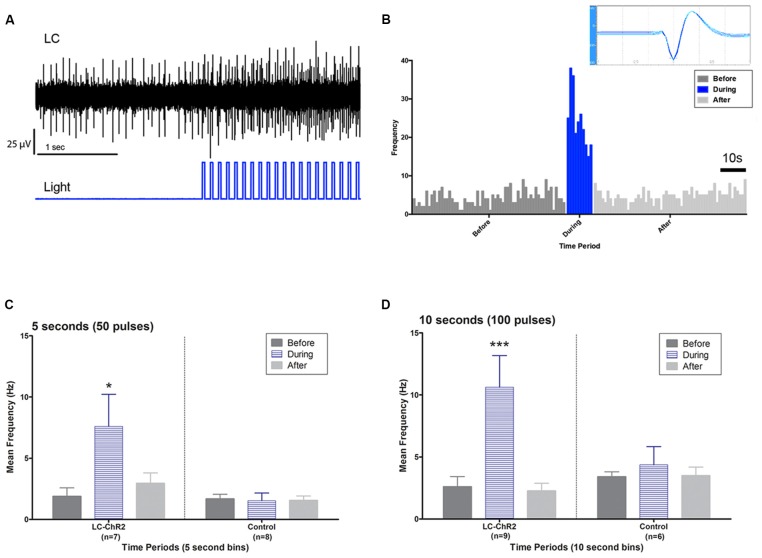
Light activation of anatomically identified LC neurons. **(A)** Oscilloscope recording showing LC units during baseline and during light pulse train activation. Note activation continuing between pulses and the recruitment of additional units. **(B)** LC unit waveform cut by Datawave algorithm (inset) and firing pattern plotted as a histogram before, during and after a 10 s light activation. **(C)** Group histograms for LC and control units during a 5 s light activation protocol. **(D)** Group histograms for LC and control units during a 10 s light activation protocol. Single asterisk, *p* < 0.05; triple asterisks, *p* < 0.001.

Optrode histology revealed that only 9 of 20 rats recorded had optrode placements in the LC (see Figure 2). Light stimulation with 5 s 10 Hz trains of 30 ms light pulses was applied in 13 rats. Of those seven had optical probes in LC and responded to the light. Optrode placements outside the LC never responded to the light and are presented as control subjects. The mean baseline firing rate (± SEM in the 5 s prior to light activation for the seven LC rats was 1.89 Hz ± 0.71, during light activation at 10 Hz, firing was an average of 7.60 Hz ± 2.87 and cells returned to 2.94 Hz ± 1.11 after light off. The control rats given the same activation protocol had a 5 s baseline of 1.68 Hz ± 0.059, and firing did not increase during the light (1.53 Hz ± 0.54), while the post light average was 1.55 Hz ± 0.55, see Figure 3. Thus, consistent with the location of channel expression, only cells within the LC showed light activation (*F*_(2,12)_ = 4.73, *p* < 0.05).

Similar outcomes were seen for the 10 s trains. Ten seconds light trains at 10 Hz were applied in 15 rats. Histology identified nine as recorded in LC (six had also been tested with 5 s trains) and six as recorded outside of LC (four also tested with 5 s trains). The 10 s baseline firing rate of the LC neurons was 2.60 Hz ± 0.87 (SEM) with an increase to 10.62 Hz ± 3.54 with light activation and a post-light frequency of 2.26 Hz ± 0.75. (*F*_(2,16)_ = 14.07, *p* < 0.05). The control baseline was 3.40 Hz ± 1.39, during light 4.37 Hz ± 1.78, and in the 10 s following optical stimulation firing was at 3.48 Hz ± 1.42, see Figure 3. Again cell firing at control locations did not change with light stimulation.

### Control and Experimental Cell Waveforms

The average waveforms selected by cluster cutting in DataWave for cell firing analyses were evaluated separately for experimental and control optrode placements. In the experimental group the average LC cell waveform in this group (*n* = 10) had an amplitude of 64.3 μV (±20.3 μV), a half width of 280 μs (±17 μs), a peak latency of 185 μs (±6.6 μs) and an asymmetry ratio of 1.44 (±0.13). The average pre-baseline frequency of the experimental condition was 3.2 Hz ± 0.9). In the control group the average cellular waveform (*n* = 10) had an amplitude of 44.3 μV (±4.5 μV), a half width of 300 μs (±26.9 μs), a peak latency of 180 μs (±11.7 μs) and an asymmetry ratio of 1.42 (±0.08). The average pre-baseline frequency of the control condition was 3.61 Hz (±.9). On the basis of waveform analysis the experimental (optrode within LC boundaries) and control (optrode outside the LC boundaries), waveforms did not differ.

### Locus Coeruleus Optical Stimulation Effects With a 10 min Light Train

**Figure 4:**
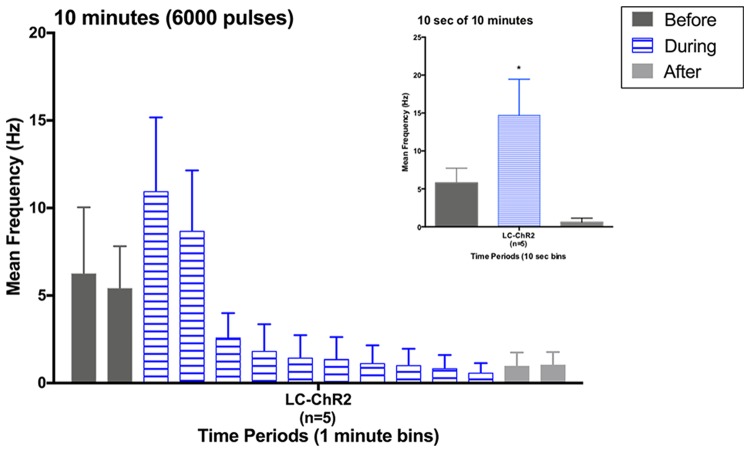
Histogram for LC units during the 10 min light activation protocol in 1-min bins. Inset: histogram for the first 10 s of light activation in the 10 min period. Note similarity to previous 10 s light data. *p < 0.05.

Five rats that had all shown the LC cell activation to 10 s light trains as exemplified in Figure 4, showed a biphasic pattern of excitation and then inhibition to LC light activation when given the sustained 10 min train of the same light pattern (see Figure 4). The average baseline for the 2 min preceding the light was 5.84 Hz ± 2.61 (SEM), which increased to 9.83 Hz ± 4.39 during the first 2 min of light stimulation. The response in the first 10 s was also the normal increase, see inset in Figure 4. In the remaining 8-min of the light-on condition there was a significant decline in LC firing to an average of 1.36 Hz ± 0.51. Firing remained lower post-light stimulation. One rat had a PP-DG evoked potential profile that changed shape over time so that for examining 10 min light effects on the PP-DG evoked potential only four rats were included. A sixth rat showed a variant light response over 10 min that increased over time. Optrode placements for all rats in the LTP experiment are shown in Figure 2.

### PP-DG Evoked Potential Modulation by 10 min Optical Stimulation

**Figure 5:**
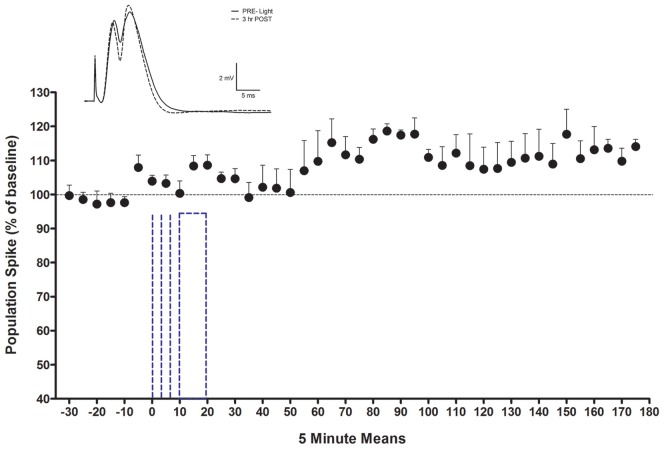
LTP of population spike for LTP rats with consistent waveforms (*n* = 4). Inset shows an average waveform during baseline and at the end of the potentiation period from one subject. Note absence of EPSP slope change. The dotted lines indicate light activation. The first three denote 5 or 10 s periods and the last denotes the onset and offset of the 10 min activation period. The LC optrode was adjusted to optimize unit recording for light activation prior to 0.

Rats (*n* = 4) in the 10 min light activation NE-LTP group had an average baseline population spike amplitude of 3.69 mV (±1.2), an EPSP slope averaging 5.8 mV/ms (±1.3) and an average latency of 4.6 ms (±0.4). The average current used for stimulation during the experiment in this group was 650 *μ*A. They exhibited a delayed and modest increase in population spike amplitude observable ~30 min following the 10 min light stimulation protocol. A repeated measures ANOVA confirmed this increase to be significant (*F*_(41,123)_ = 2.57, *p* < 0.0001), see Figure 5. The average maximal increase for these rats was 126%±5.3. The percentage increase was similar for the rats given a 50% maximal current (*n* = 2) and a maximal current (*n* = 2) for their baseline PP pulses. There were no significant changes in EPSP slope or population spike latency. The occurrence of the spike on the rising portion of the EPSP slope and a latency to spike peak of less than 5 ms suggests a medial PP population spike (Bramham et al., 1991) was evoked.

### PP-DG Evoked Potential Input-Output Curves Pre and Post 10 min Light Stimulation

**Figure 6:**
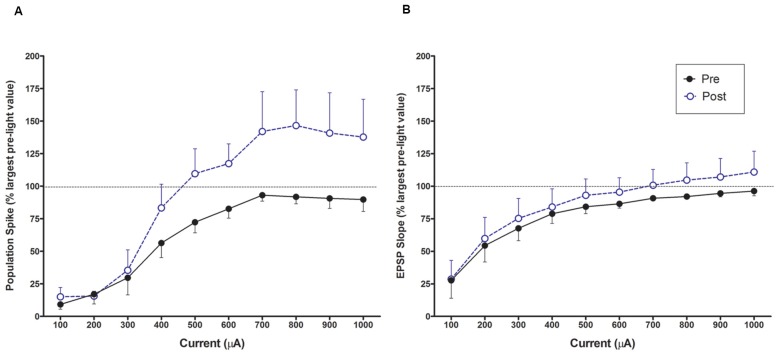
Input output curves for increasing perforant path currents prior to light activation LTP baseline and after the conclusion of the experiment. **(A)** A leftward shift for population spike occurs after the experiment for population spike amplitude once threshold is exceeded. **(B)** There is no change in EPSP slope input/output over the same period.

The population spike amplitude was increased over pre-light (baseline) measures for all current intensities higher than 300 μA. A 2 × 10 within ANOVA found a significant interaction between pre/post and current *levels* F_(9, 27)_ = 2.880, *p* < 0.05; see Figure 6). There was a leftward shift in responses at the end of the experiment confirming an increase in population spike amplitude to the same current intensity following the 10 min LC stimulation protocol. There were no changes in EPSP slope or population spike latency in the input output curves in comparing beginning and final values.

### PP-DG Evoked Potential Paired Pulse Stimulation Effects Pre and Post 10 min Light Stimulation

**Figure 7:**
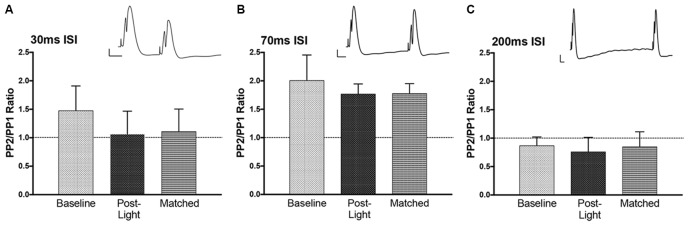
Paired pulse effects at three interstimulus interval (ISI). Example paired pulse waveforms for each interval at baseline are included. Bars = 2 mV, 10 ms. **(A)** At 30 ms ISI prior to the experiment (Baseline) there was no clear inhibition. This was similar at the end of the experiment (Post-Light) and also seen when P1 sizes were matched by lowering the current (Matched). **(B)** At 70 ms ISI the expected potentiation occurred land this was unchanged at the end of the experiment. **(C)** At 200 ms ISI the expected inhibition was observed, but again there was no effect on this paired pulse inhibition of the light manipulation and the enduring NE-LTP.

*T*-tests were performed on each paired pulse interval data set. Paired pulse ratios were averaged for to determine if the intervals resulted in inhibition (PPR <1) or facilitation (PPR > 1). The average ratios for population spike amplitude at baseline were not significantly different from 1 (Figure 7), but the direction of effect was appropriate at 70 ms (facilitation) and 200 ms (inhibition; Joy and Albertson, 1993). There was no evidence of inhibitory feedback at 30 ms, which suggests that interval was too long to engage rapid GABA-A inhibition. Following NE-LTP the increase in P2 at the 70 ms paired pulse interval was significant, but the direction of effect (facilitation) was unchanged, suggesting a reduction in variability of the predicted response at this interval. This significant effect was seen whether the post-LTP P1 potentials were matched to the pre-LTP baseline with current reduction (matched condition) or whether current was unchanged.

EPSP slope P2/P1 ratios at baseline (not shown) were 0.86 (±.03) for ISI 30 ms, 0.90 (±.08) for ISI 70 ms, and 0.81 (±.07) for ISI 200 ms. These ratios were similar post NE-LTP with both matched and unmatched P1s. The mild inhibition seen at 30 and 200 ms ISI was significantly (*p* < 0.05) smaller than in both pre and post LTP testing. This suggests reduction in transmitter release at these two intervals.

These profiles for both population spike and EPSP slope suggest no effect of NE-LTP more than 3 h post-induction on baseline local circuit modulation at the three paired pulse intervals assessed.

### LC-PP Evoked Potential Effects for Single Pulse LC Light Pulses as a Function of the LC-PP Interval

**Figure 8:**
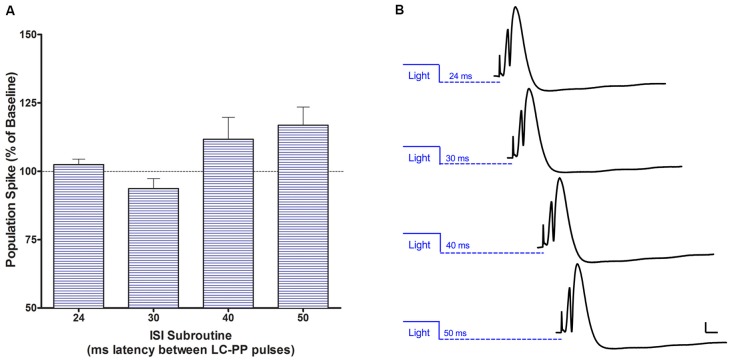
**(A)** Acute single population spike potentiation when a 50 ms LC light pulse is delivered 40–50 ms prior to the perforant path pulse. This single pulse effect is consistent with conduction times for LC fibers. **(B)** Examples of modulatory effects of single LC light pulses preceding PP input at each of the four intervals tested. Bars = 2 mV; 5 ms.

Using a 30 ms light pulse 24, 30, 40 or 50 ms prior to the PP pulse produced no significant modulation of population spike amplitude or EPSP slope. However, a repeated measures ANOVA (*F*_(3,9)_ = 3.982, *p* < 0.05) was significant when a 50 ms light pulse was given prior to the PP pulse with a significant quadratic trend (*F*_(1,3)_ = 13.828, *p* < 0.05) reflecting increased population spike amplitude at 40 and 50 ms after the light pulse, see Figure 8. The largest increase was 117% ± 7.6. There was no modulation of EPSP slope.

## Discussion

### Anatomical Observations

Co-localization was assessed in 20 rats and ChR2 channels were observed in more than 70% of the DBH neurons. These numerical data are consistent with earlier optogenetic images for rats, in which an overshadowing of DBH staining by ChR2-EYFP is also occasionally observed (Hickey et al., 2014; Takeuchi et al., 2016). These outcomes are similar to the range of co-localizations observed in mouse images (see, for example Kempadoo et al., 2016; Soya et al., 2017; Li et al., 2018), but lower than two reported counts in mice (>90%; Carter et al., 2010; Takeuchi et al., 2016).

There was a range of times after infusion before sacrifice in these experiments. The five rats with 2 months or less incubation time to co-localization assay were compared with the five rats with 5.5 months or more incubation time. There was no difference in average co-localization (~63%) or in average LC neurons observed per section (846.5). This suggests channel expression was consistent and well-maintained over time as reported earlier (Zhang et al., 2010). There were also no significant correlations between incubation time and co-localization or cell numbers/section.

In the majority of LC images obtained in our series, we observed ChR2-eYFP expression in a group of cells between the central LC and the 4th ventricle (see Figure 1 for examples). Felten and colleagues (Burnett and Felten, 1981; Felten et al., 1981) identified these cells as LC projecting tanycytes. LC tanycytes locally line the ventricular wall and send their processes deep into the LC where they make contact with DBH cell bodies and dendrites. It was initially proposed that the tanycytes take up substances from the CSF for transport to monoamine neurons.

In avian LC, LC tanycytes take up both nerve growth factor and urotensin-1 (Feng et al., 2011). Nerve growth factor is transferred into LC neurons, which then transport it along their axons to target structures. This transcytosis may be mediated *via* specialized synaptoid contacts. Ghrelin in mice is also taken up by LC tanycytes (Cabral et al., 2013). Feng proposed the LC tanycyte pathway provides a route for CSF to selectively modulate LC function and, possibly, vice versa.

Our images support the proposed reverse transport of materials from LC cells to tanycytes, since tanycytes themselves could not express the DIO-controlled ChR2-eYFP gene. ChR2-eYFP is larger than the peptides that have so far been studied, but ChR2 subunits are smaller, and could be transcytosed. Images similar to ours for ChR2-eYFP expression in the LC tanycyte region can be observed in other ChR2-eYFP LC optogenetic studies (mice, Soya et al., 2017; Figure 6C; Kempadoo et al., 2016; Figure 2A; rat, Hickey et al., 2014; Figure 1A). Collectively, these images are consistent with the hypothesis that LC may export to, as well as receive material from, its tanyctyes.

LC is selectively vulnerable to early neurodegenerative changes that may initially be environmentally/CSF mediated (Itzhaki et al., 2016; Mather and Harley, 2016) and might have a role in secreting soluble hyperphosphorylated tau back to the CSF (Mufson et al., 2016; Pascoal et al., 2017) as well as in its transport to entorhinal cortex and other modulatory cell groups (Braak and Del Tredici, 2011). Our LC-co-localization images suggest studies targeting LC-tanycyte function may be of value in understanding these effects.

### Locus Coeruleus Optogenetic Cell Activation Effects *in vivo*

Our work with LC optogenetic recording revealed three unexpected features of such recording *in vivo*. First, using standard approaches to position optrodes near LC neurons for recording were only partially successful as these approaches also selected for non-LC cells. Second, we assumed from *in vitro* optogenetic activation studies that we would have strong temporal control of LC firing *in vivo* as well. Multiunit recording suggests this is not the case. Third, we assumed that having established consistent light pulse activation for 5 and 10 s periods, light activation would continue to be effective over minutes. This was also not the case. We will consider each point in turn.

First, of the 20 rats thought to have optrode placements in the LC based on stereotaxic co-ordinates, slow firing rates and a response to foot/tail pinch, only 10 had cell placements that were driven by optogenetic activation. The rats with successful activation had optrodes located in the LC (now called the experimental group), while those that did not show light responses did not have LC optrode localization (control group). Both groups had good LC channel expression. Placements for the optrodes of the two groups were shown in Figure 2.

Thus, despite the fact that all cells met the putative electrophysiological criteria used here for LC neurons during recording and that *post hoc* waveform analysis did not differentiate the control cells from the LC cells, histological analysis of optrode tip placement revealed two groups: cells within the LC nucleus and those outside it. This argues that one cannot assume slow-firing, pinch-responsive cells at the approximate level of the LC are LC neurons. Importantly, consistent with the spatial selectivity of optogenetics, non-LC neurons did not respond to light activation.

Second, while 5 and 10 s of a 10 Hz train of 30 ms pulses of light activation changed LC firing from an average of ~1.9–2.5 Hz (see Takeuchi et al., 2016 for a similar LC baseline in awake mice) to 7.8–10.6 Hz and might be taken as evidence for near 10 Hz driving by the trains, as *in vitro*, that was not our impression. As seen in Figure 3, LC activation responses in our experiments occurred over a wide range of frequencies and were not synchronized to the light pulses (see below for other examples *in vivo*).

*In vivo* optrode studies are relatively rare in LC. The first LC optogenetic study in mice (Carter et al., 2010) verified light activation in juvenile LC slices using patch recording, reporting one-to-one light pulse to action potential driving, although neurons could not be driven beyond 20 Hz (see also Hickey et al., 2014 in rat) where the driving limit was 10 Hz). Mouse studies since 2010 have typically also provided indirect evidence of LC neuron activation, using either, for example, slice recordings (Wang et al., 2014), post mortem *cfos* activation (Uematsu et al., 2017; Li et al., 2018), NE/DA release in a target structure (Kempadoo et al., 2016) or desynchronized EEG (Kim et al., 2016) as indicators. Temporal correlation of light activation to behavior change provides another class of indirect evidence, usually demonstrating that behavioral changes vary with light activation parameters (Soya et al., 2017; Glennon et al., 2018).

An exception among mouse studies is the report of McCall (McCall et al., 2015) who undertook *in vivo* LC optrode recording in a separate group of mice. They found significant increases in population firing rates using 5 Hz 10 ms light pulses for 20 s but again firing increases differed widely among individual neurons and none fired at 5 Hz in the activation period. The differences were explained, in part, by initial baseline firing, with cells more active at baseline being more responsive to light and then firing beyond the driving rate. Other LC cells, which did not appear directly photosensitive, as they failed to respond during the light pulse itself, also increased their firing rate, possibly due to coupling with light-activated neurons. The McCall results suggest one-to-one light pulse action potential driving does not occur in LC *in vivo*. Rates instead are modulated by an LC neuron’s propensity for firing, by its proximity to light spread, and by activity in neighboring neurons.

Third, we failed to see sustained LC activation when our train was extended to 10 min from 10 s This unexpected result finds support in earlier reports. In experiments using rat LC neurons and optogenetic activation, Hickey et al. (2014), found, both *in vitro*, and in a heart-brainstem preparation with intact brainstem networks, that short periods of pulse driven LC opto-excitation were followed by periods of LC silence depending on the duration and intensity of train activation. Our average light current (60 mW) was twice that used by Hickey et al. (2014), although the pulse durations and the optogenetic channels expressed are similar. Our intensity is more likely to generate periods of LC silence since depolarization itself is likely to be the critical variable. Recent *in vitro* experiments have revealed that LC neurons counter depolarizing input with strong activation of intrinsic hyperpolarizing calcium-mediated potassium currents that curtail firing (McKinney and Jiang, 2018). *In vivo*, in addition to Hickey’s observations in rat LC, the first optogenetic LC activation study in mice reported a significant reduction in cortical NE from LC fibers of train-activated LC neurons when using trains above 5 Hz (Carter et al., 2010), an outcome consistent with LC silencing during the 10 Hz train here. McCall et al. (2015, Figure 2H, in rat) also show examples of LC accommodation to optogenetic activation, and of increasing inhibition of LC firing with repeated 20 s 5 Hz trains.

Besides the new report that depolarization recruits intrinsic hyperpolarizing currents in LC neurons, other mechanisms have been identified that curtail LC firing. Specifically, local release of NE in LC activates inhibitory LC α2-adrenergic receptors (Aghajanian et al., 1977) and late developing inhibition with LC activation *via* somatodendritic LC α_2_-adrenergic receptors has been reported in multiple studies (Svensson et al., 1975; Huang et al., 2012). Second, post-activation inhibition driven by an inhibitory AMPA receptor on LC neurons has been shown to be recruited ~2 min after initial AMPA activation (Zamalloa et al., 2009). This mechanism is interesting because of its time frame, but it is not clear if glutamate is increased locally in LC under our conditions.

As a caveat it should be acknowledged that loss of LC recording could be an issue. Local changes in blood pressure or other variables could cause the loss of LC cell monitoring. This seems unlikely in our five experiments as we observed stable multiunit firing over 10 min. We found that light-activated firing for each of the five LTP rats with unit recording was similar in the first 10 s light pulse trial to the first 10 s of the 10 min light train given more than 10 min later. This argues for relatively stable recording. Given the replicated finding of induced LC silence 2 min after train onset, when LC activation was assumed, it is evident that optrode recordings in *in vivo* optogenetic experiments will be important for accurately interpreting the relation of LC firing patterns to LC functional effects.

### PP-DG Evoked Potential Modulation

Using single pulse light activation of LC neurons at specific intervals prior to a PP pulse revealed that population spike potentiation, but not EPSP slope potentiation, could be induced with 50 ms pulses ending 40–50 ms prior to the PP pulse. There was no carry over of the potentiation to subsequent spikes. The spike potentiation effect required strict timing and a longer light pulse than that used for the other experiments (30 ms). The use of both 30 and 50 ms pulses for the single pulse experiments was based on the report (Hickey et al., 2014) that the charge times of rat LC neurons for the channels we used is relatively long and that 50 ms or longer pulses improve spike reliability.

The LC-PP potentiation intervals that we observed are consistent with conduction times estimated from hippocampus and cortex for unmyelinated fibers of rat LC (Nakamura and Iwama, 1975). Electrical LC activation prior to PP pulses also produces single PP-spike potentiation between ~40 (Assaf et al., 1979; Ramírez and Carrer, 1983) and ~50 ms (Dahl and Winson, 1985). Potentiation was larger in these earlier studies with electrical stimulation (117% here vs. 140% earlier), but this may relate to the relatively small volume of LC that our light cone would have reached and the ~62% level of channel co-localization. Using those parameters, we estimate less than 20% of LC neurons would have been engaged by our dorsally located optrodes, although recruitment of neighboring LC neurons would increase that percentage and did appear to occur (see Figure 4 oscilloscope recording).

The finding of LC mediated single pulse potentiation of glutamate PP input argues for an acute LC mechanism for informational input enhancement that is tightly controlled temporally. The transient single pulse effect is different from the more widely studied phenomena of LC-NE priming of learning and memory that can be generated by LC activation manipulations before (Ballarini et al., 2009; Moncada et al., 2011; Viola et al., 2014) or after the learning event (Takeuchi et al., 2016), and with LC’s ability to generate a delayed long-lasting PP-evoked potential potentiation (NE-LTP).

We replicated delayed long-lasting NE-LTP of the PP population spike in DG using 10 Hz optogenetic activation of the LC for only a 2 min period (nominally 10 min). The generation of spike NE-LTP in DG depends on the NE increase measured in DG (Harley et al., 1996). A slow onset of spike potentiation relative to the time of LC activation here parallels what has been observed with glutamate LC activation in awake rats (Walling and Harley, 2004), and with orexinergic LC activation in urethane-anesthetized rats (Walling et al., 2004), although more rapid onsets have also been *in vivo* (e.g., Harley and Milway, 1986) and *in vitro* (e.g., Stanton and Sarvey, 1987). Nicotine activation of LC, which is rapid, induces a slow developing potentiation of medial PP EPSP slope that is blocked by silencing the LC (Rajkumar et al., 2013, 2017). Larsen and Redt report continual vagal nerve stimulation also induces late appearing PP-spike potentiation (Larsen et al., 2016). Finally, slowly developing LC-NE potentiation of Schaeffer collateral (SC) EPSP currents in CA1 occurs when CA1 NE axons are optogenetically activated with three trains of 60 brief pulses over a 5 min period (Takeuchi et al., 2016).

Another observation during the PP-evoked potential recording is also of interest. A transient enhancement of population spike amplitude can be observed in the 5 min block preceding the onset of the light activation protocol (0 time point in Figure 5). To optimize the monitoring of LC neurons during the light activation protocol, small changes were made in the stereotaxic localization of the optrode assembly in this interval for each rat. We suggest that direct mechanical activation of LC neurons is the most likely candidate for the mediation of the enhancement seen in this block. LC cfos-indexed activation with mechanical cannula movement among LC neurons has previously been reported (Stone et al., 1995).

The glutamate amplification of NE (GANE) hypothesis links the level and nature of local glutamate events with the level of local NE (“NE hotspots”) and accounts for the varying NE effects on plasticity of glutamate inputs, which can range from NE-LTP to NE-LTD depending on the glutamate and NE inputs and patterns (Mather et al., 2016). As predicted by GANE, a conjunction of LC and PP inputs is required for the enduring PP plasticity effects of LC activation (Reid and Harley, 2010). NE and glutamate levels in DG have recently been shown to interact directly in the successful production of active avoidance learning (Lv et al., 2017). The recruitment of plasticity proteins and epigenetic changes by LC-NE/DA (Moncada et al., 2011; Maity et al., 2015, 2016; Martins and Froemke, 2015) accounts for delayed plasticity and metaplasticity effects when LC activation occurs either prior to, or following, activation of glutamatergic input. We suggest optogenetic LC activation engagement of plasticity proteins supported the late expression of enduring spike potentiation to the continually repeated glutamate PP input.

GANE also explains the new results of Takeuchi in CA1 where strong optogenetic CA1 NE fiber activation produces a delayed and enduring potentiation of SC synaptic input, while a weaker optogenetic train of CA1 NE fiber activation had no effect on SC input *unless* combined with a weak theta tetanus of the SC input. In the latter case, the weak optogenetic activation protocol significantly increased and prolonged SC potentiation.

In DG, Hansen and Manahan-Vaughan (2015a) observe LC-NE/β-adrenergic dependent potentiation of a weak high frequency PP input to DG following novel holeboard exploration that naturally activates LC. We have also observed holeboard exploration-induced potentiation of single PP inputs that are β-adrenergic dependent consistent with the LC bursts recorded during novelty exploration (Kitchigina et al., 1997). Manahan-Vaughan, however, finds only LTD when intermittent PP input is paired with electrical LC stimulation (Hansen and Manahan-Vaughan, 2015b), while we consistently see LTP of PP input when using continual PP single pulse pairing. These differing results can now be assessed in the context of GANE by directly varying glutamate input patterns, while holding NE input constant or, conversely, by holding glutamate input constant and varying NE input. Adding to the subtlety and complexity of NE neuromodulation in DG, medial and lateral PP inputs compete for potentiation and either pathway can win depending on firing patterns (Edison and Harley, 2012). GANE is an account of the competitive interactions among informational inputs for dominance in cognitive processes.

The present data are congruent with GANE and with the likelihood that the levels and patterns of local NE and glutamate release determine the timing, the magnitude and even the direction of network plasticity that occurs. It will be of interest to explore these critical parameters with optogenetic methods in hippocampus and in other LC target systems. Recent studies further suggest modularity in LC connectivity (Chandler et al., 2014; Chandler, 2016; Uematsu et al., 2017), a facet readily explored with retrograde uptake of optogenetic channels and with the use of varying optrode diameters and their placements in LC. Of particular interest for better understanding the role of the LC in learning and memory is the recent report that optogenetic pairing of LC with activation of auditory tones leads to an enduring enhanced response of LC input to those tones (Martins and Froemke, 2015). The PP system offers an opportunity to ask if the pairing of PP input with optogenetic LC activation also alters the influence of PP inputs on LC firing itself during NE-LTP.

## Author Contributions

MQ carried out the experiments described here. VS provided technical assistance. DS, GM, CH and SW assisted with hypothesis development, experiment selection, data analysis, and theoretical interpretation. MQ, DS, GM, CH and SW contributed to the written article. MQ was supervised in this research project as an MSc student by SW.

## Conflict of Interest Statement

The authors declare that the research was conducted in the absence of any commercial or financial relationships that could be construed as a potential conflict of interest.
